# Recurrent Subglottic Stenosis in a 16-Month-Old Male in the Setting of Influenza A, Intubation, and Honey Consumption

**DOI:** 10.7759/cureus.52315

**Published:** 2024-01-15

**Authors:** Allison T Jakiel, Krisdaniel Berreta, Hanna S Sahhar, Sami Rishmawi

**Affiliations:** 1 Pediatric Intensive Care Unit, Edward Via College of Osteopathic Medicine, Blacksburg, USA; 2 Pediatric Intensive Care Unit, Edward Via College of Osteopathic Medicine, Spartanburg, USA; 3 Pediatric Medicine, Edward Via College of Osteopathic Medicine, Spartanburg, USA; 4 Pediatric Intensive Care Unit, Spartanburg Regional Healthcare System, Spartanburg, USA

**Keywords:** pediatric intensive care unit (picu), post-intubation complications, post intubation tracheal stenosis, honey consumption, recurrent tracheal stenosis, pediatric influenza a, subglottic stenosis

## Abstract

Recurrent episodes of subglottic stenosis are rare in the literature, and the etiologic causes are misunderstood but can be congenital, idiopathic, or iatrogenic in nature. Complications of intubation can result in subsequent inflammation and reactive processes. This case involves a 16-month-old male who suffered from a recurrent episode of subglottic stenosis in the setting of croup, influenza, and honey consumption. He had presented to the emergency department in respiratory distress after ingesting a home remedy of onion juice and honey. He had been discharged one day prior from the pediatric intensive care unit after four days of intubation and a seven-day hospital course with evidence of croup on imaging. He was readmitted, and subglottic edema and narrowing were confirmed via endoscopy, which prompted antibiotic treatment and close monitoring. After three days of monitoring and re-evaluation by bronchoscopy, the patient's condition began to improve, and no intubation was necessary. It is unclear what the cause of recurrent subglottic stenosis is due to the patient's clinical picture being clouded by a potential allergic reaction to honey versus an inflammatory reactive process post-intubation from the previous admission days prior. This case emphasizes the need for further research on the prevalence and etiology of recurrent subglottic stenosis and a deeper understanding of how to optimize diagnosis and treatment.

## Introduction

This case report was previously displayed as a poster at the American College of Osteopathic Pediatricians 2023 Spring Conference on April 29, 2023, in Louisville, KY.

Subglottic stenosis is the narrowing of the area between the vocal folds and the inferior border of the cricoid cartilage. It can be congenital, acquired, or idiopathic, which is often misdiagnosed as asthma [1]. Symptoms include biphasic stridor, dyspnea, and intercostal, suprasternal, and abdominal retractions [2]. Honey is often used to reduce asthma-related symptoms, and in this case, it was used as a therapeutic means; however, the etiology of subglottic stenosis is not fully understood [3]. Subglottic stenosis could be acquired post-intubation, infection, inflammation, tumors, ingestions, and trauma [4].

In cases of post-intubation, pressure necrosis can occur due to the endotracheal tubing and can lead to mucosal edema and ulceration [5]. This inflammation causes increased mucus production, predisposition to bacterial overgrowth, type II collagen thickening, and damaged ciliate epithelium. The growth of scar tissue, mucosal sloughing, and granulation ultimately results in intraluminal narrowing of the upper airway and subglottic stenosis. Delayed stenosis occurs when this process is prolonged [6]. Over 24-36 hours after extubation, reactive edema, mucosal damage and obstruction, retractions, and stridor can be evident. It will typically worsen before improving, warranting close monitoring of patients. After 72 hours, if the stridor persists or has a new onset, the airway is severely affected, prompting endoscopic evaluation and intervention [7].

In 2013, a prospective study indicated an incidence of 11.38% of subglottic stenosis in 123 children post-intubation [8]. Subglottic stenosis can be evaluated via bronchoscopy and imaging. Subglottic stenosis is typically managed through endoscopy with either the removal of obstructing granulation tissue, steroid injection to reduce the inflammation, or balloon dilation to widen the airway [9]. Placement of a tracheotomy or laryngotracheal reconstruction may also be considered in more severe cases where the cartilage is compromised or there is congenital subglottic stenosis [7].

The goal of this case report is to highlight a unique presentation of recurrent subglottic stenosis in the setting of Influenza A infection, croup, intubation, and honey ingestion resulting in a subsequent immune reaction.

## Case presentation

**Figure 1:**
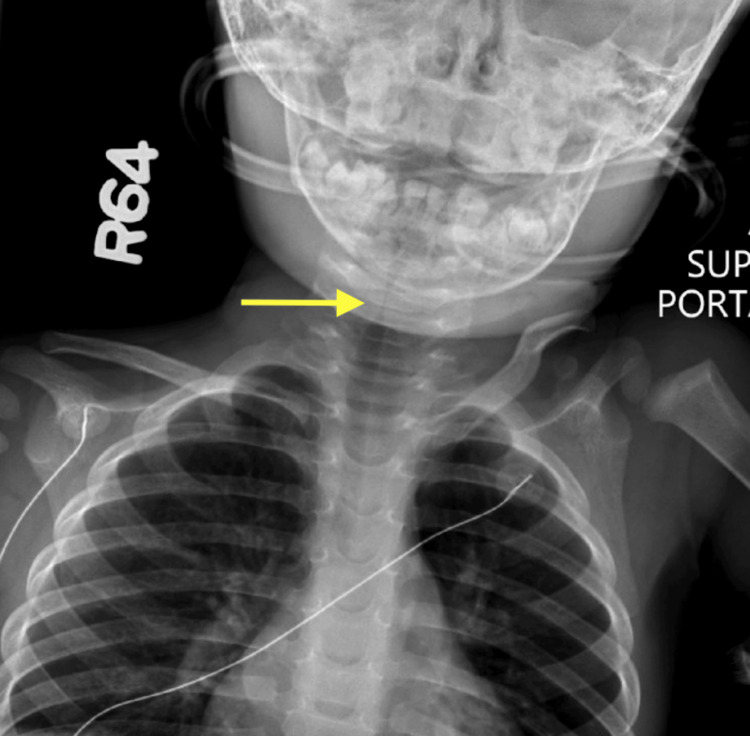
Posterior-anterior radiograph showing initial subglottic stenosis and diffuse tracheal narrowing

**Figure 2:**
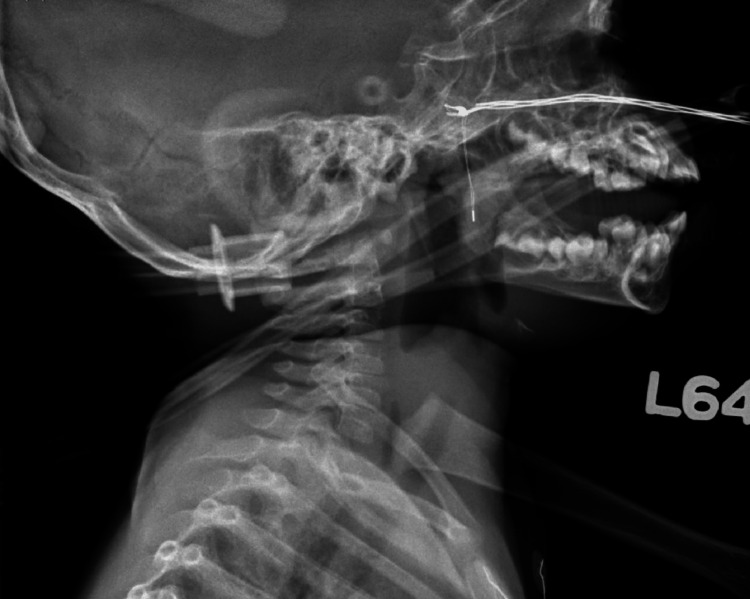
Lateral radiograph showing initial subglottic stenosis and diffuse tracheal narrowing

**Figure 3:**
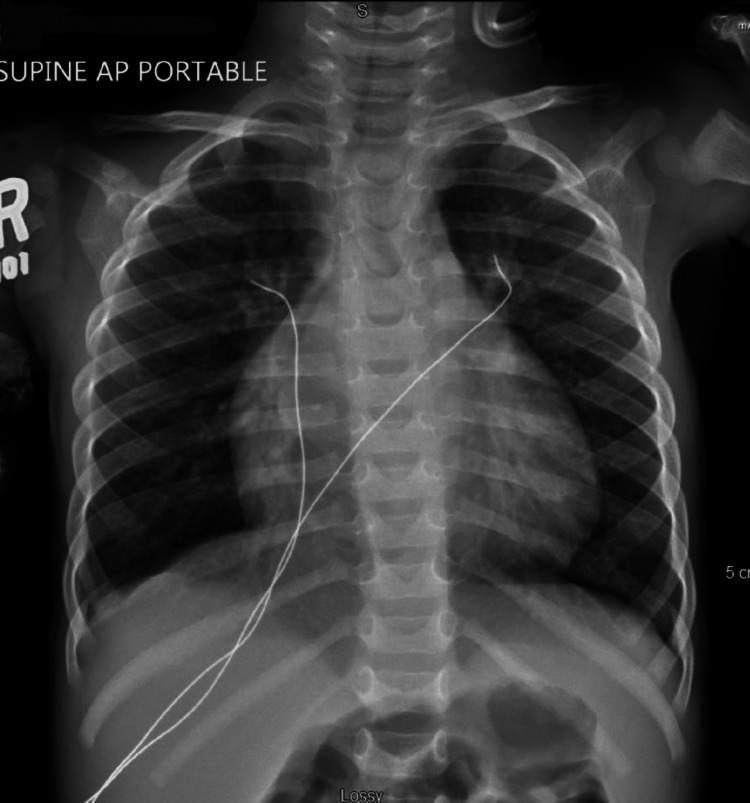
Chest radiograph for initial pediatric intensive care unit (PICU) admission

**Figure 4:**
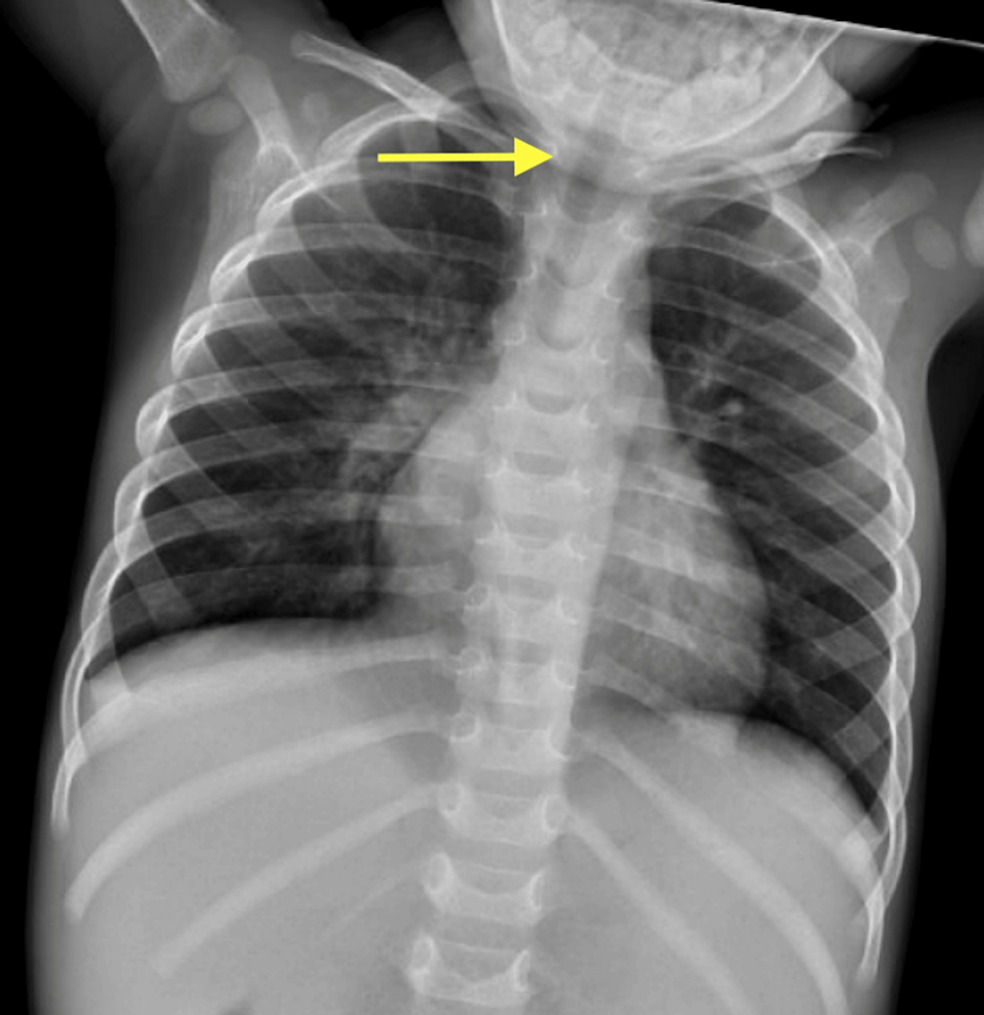
Chest radiograph on day of discharge from initial pediatric intensive care unit (PICU) admission showing clinical improvement of stenosis

A 16-month-old African American male presented to the emergency department (ED) with a primary concern of lip edema approximately 30 minutes after consumption of a honey and onion juice home remedy. This was the patient’s second occurrence of an immune reaction following honey consumption. The patient had been discharged the day prior from the pediatric intensive care unit (PICU) after a seven-day hospital course and four days of intubation due to subglottic stenosis and diffuse tracheal narrowing (Figures 1-4) secondary to croup in the setting of croup secondary to influenza A. According to his mother, he had been improving overall but began to have trouble breathing and a barky cough. He had recently been prescribed albuterol by an urgent care physician who had diagnosed the patient with influenza the week prior after presenting due to lip swelling and respiratory distress. On examination, the patient was febrile at 100.3 F, tachypneic at a rate of 35, heart rate of 162, audible stridor, intercostal and supraclavicular retractions, and abdominal breathing.

The patient was born by c-section at 39 weeks with no significant prenatal or neonatal history. He was 19.88 in, 7 lb 9 oz, with Apgar scores of 7 and 9 at birth. The patient had a known allergy to lactose. He consumes a normal diet, is up-to-date on all vaccinations, and regularly follows up with a primary care physician. The patient’s medical history was only significant for mild developmental delay, an underweight BMI of 13.05 kg/m2, exposure to third-hand/second-hand smoke, and eczema. He lives at home with his mom, dad, and older sister. His family history is positive for hypertension in his mother and several family members with eczema without any known cases of atopy or asthma.

**Figure 5:**
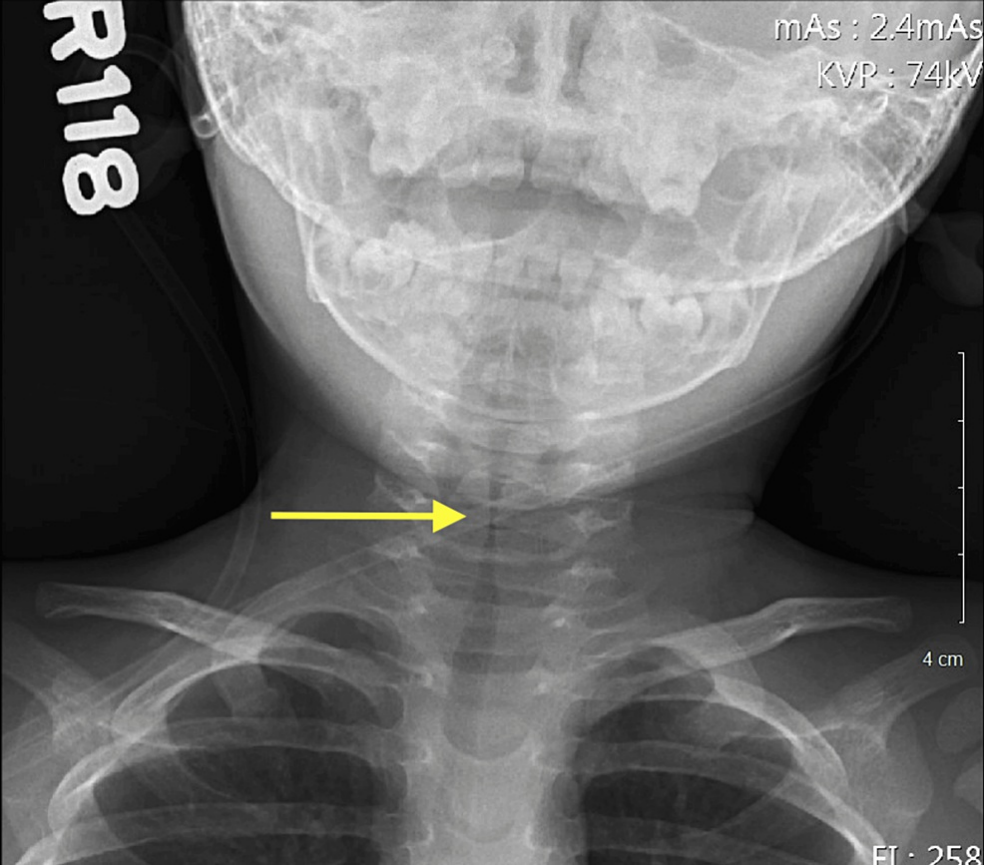
Posterior-anterior radiograph showing recurrent subglottic stenosis and diffuse tracheal narrowing at second pediatric intensive care unit (PICU) admission (one day after discharge from Initial PICU)

**Figure 6:**
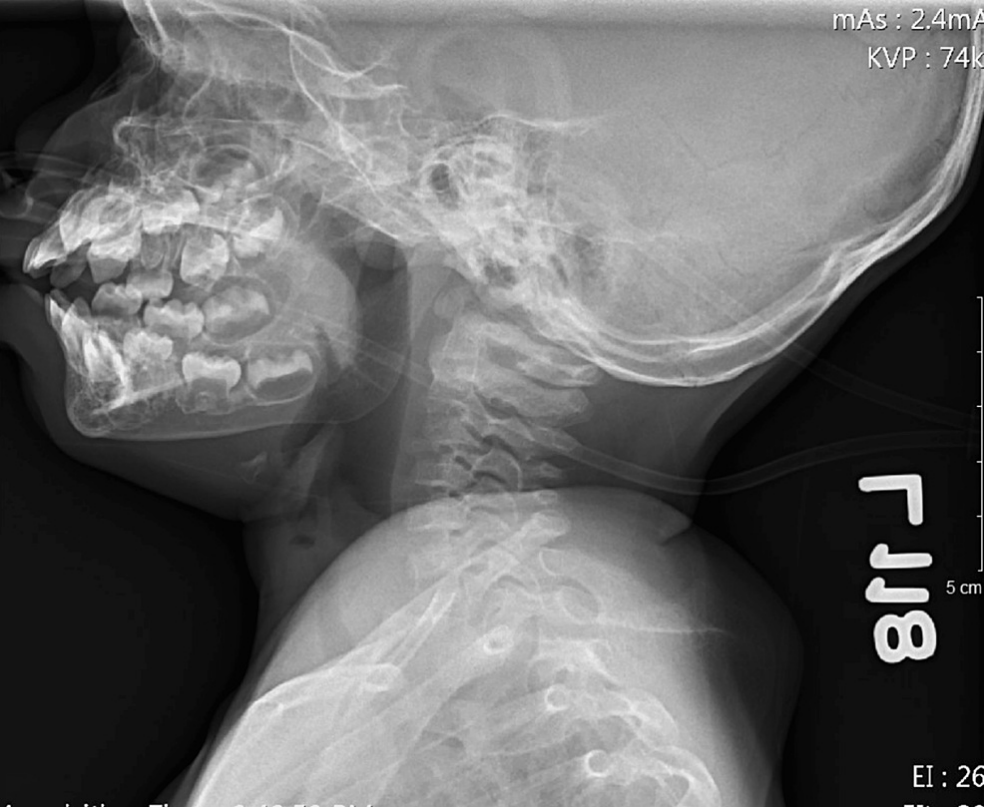
Lateral radiograph showing recurrent subglottic stenosis and diffuse tracheal narrowing on second pediatric intensive care unit (PICU) admission (one day after discharge from initial PICU)

In the ED, he received 5 L/min of oxygen via nasal cannula. Given his recent PICU admission and intubation, the patient was admitted due to persistent respiratory distress and exacerbations. The patient was given a regimen of budesonide (Pulmicort) 0.25 mg/2 mL nebulizer solution, dexamethasone IM 5 mg, Benadryl IM 11 mg, epinephrine IM 0.1 mg, Racepinephrine 2.25% nebulizer solution, and sodium chloride 0.9% bolus pre-mix to reduce airway inflammation and the severity of the exacerbation. The patient was found to have wandering, roving eye movements, hypoactivity from baseline, refusal to stand, spontaneous movement of all four extremities, and decreased reflexes in the lower extremity without clonus. According to his mother, the patient had been able to bear weights and walk around objects earlier that day. The current state of distress and hypoxia could be a possible etiology for the altered sensorium. Radiographs revealed subglottic stenosis and diffuse tracheal narrowing (Figures 5-6).

On day 1 of re-admission, ENT evaluated the patient at bedside via a diagnostic flexible laryngoscopy and found him to have significant subglottic edema and secretions with difficulty to discern if tracheitis was present. There was no indication for an inpatient surgical intervention, and a recommendation was made to begin broad-spectrum antibiotic coverage for possible tracheitis. The patient was treated with 200 mg of ampicillin-sulbactam every six hours, a Ciprodex nebulizer, and a dexamethasone taper. He was reevaluated by bedside bronchoscopy on day 2, which revealed an open but narrow subglottic airway, improvement in the volume of secretions, a continued deficit in the ability to clear secretions, and vocal cord hypomobility. No endoscopic images were recorded during both ENT procedures. Due to neurological symptoms, a non-sedated MRI was attempted but unsuccessful.

On day 3 of re-admission, the patient’s activity and mental status significantly improved. The patient cried for the first time since re-admission, with improvements in alertness and activity. On day 4, the patient was then transferred to the general pediatric floor and continued to receive physical, occupational, and speech therapy. He made significant improvements and was able to move all extremities, and normal strength returned. On day 5, the patient was able to safely discharge home. He was placed on a mechanical soft diet with thickened liquids and thin liquids for medications, a five-day course of amoxicillin-clavulanate, and was given an EpiPen due to the concern for honey allergy and/or anaphylaxis. Additionally, he was referred to outpatient occupational therapy, physical therapy, and speech therapy for continual support in recovery and development. To date, the patient is recovering without any further known admission for recurrent subglottic stenosis.

## Discussion

This case of recurrent subglottic stenosis was confounded by a subsequent reaction following honey consumption and multiple hospitalizations. The communication between both PICU teams to avoid intubation given the patient's airway edema and structural anatomy proved to be critical as it likely prevented additional inflammation to the airway and further removed further exacerbation of respiratory distress. This effective communication is imperative for optimal patient care across multiple providers and healthcare facilities, particularly with patients who are re-admitted into the hospital setting, as it raises concern as to why they have re-met medical admission criteria. In this case, the initial differential diagnosis during the patient's first admission was infection due to the presence of both croup and influenza. However, upon re-admission, the differential had been expanded due to concern for neurologic and immunologic etiologies in light of the patient's presentation.

Upon re-admission to the PICU, there was a concern for botulism due to the patient's decline in baseline motor skills and an abnormal neurological exam consisting of generalized hypotonia, hyporesponsiveness, and decreased lower extremity reflexes. Fortunately, the patient's physical exam improved after administration of the steroid and nebulizer regimen, and throughout the hospital course, the patient improved without any indication for a specified treatment or anti-toxin to treat botulism. This suggested that the patient's delirium, or abnormal state of sensorium, was likely present in light of the patient's hypoxic state and respiratory distress. Another possible etiology to the patient's presentation that was deemed relevant was the consumption of honey, which may have caused an allergic systemic reaction in light of the subsequent symptoms that occurred after ingestion. In type I hypersensitivity reactions, exposure to an antigen such as honey can cause either an asymptomatic or minor reaction due to an immunoglobulin E-induced release of antibodies against the antigen, which can lead to anaphylaxis or atopy with subsequent exposure [10]. This could explain why the patient’s first consumption of honey led to lip swelling but no significant change in respiratory status, but the second consumption, in the setting of respiratory compromise, resulted in an apparent and rapid change in respiratory status. While the patient lacked urticaria or hives, it is possible his dark complexion and lack of consideration for a honey allergy contributed to the non-observation of any mild skin changes. The coincidental timing of increased respiratory distress around the time of honey consumption is also plausible. This case further emphasizes the importance of a strong medical history interview during an evaluation of a recently extubated pediatric patient.

As discussed, acquired subglottic stenosis post-extubation is seen in roughly 11.38% of children, which explains the necessity of why this patient was closely monitored following extubation; however, within 48 hours of post-extubation, this patient's stridor was only present with agitation with no retractions and was improving. Between 48 and 72 hours, the stridor worsened, and biphasic stridor was noted after he consumed honey at home, which raises concern for understanding the likely etiology. This case reveals the importance of closely monitoring post-intubation for further respiratory deterioration, retractions, and stridor to reduce re-admission, reintubation, and clinical worsening. Methods to monitor include reassessing oxygen saturation for hypoxia, making notes of mentation, and any signs of distress on the physical exam and general appearance. In addition, once medically discharged, patients with known episodes of subglottic stenosis should be monitored in the long term for reoccurrences.

## Conclusions

This case report highlights a unique presentation of recurrent subglottic stenosis in a 16-month-old African American male in the setting of croup, influenza A, and honey consumption. The case emphasizes the clinical implications of continuity of care, effective communication between providers, and the need for additional research on the prevalence and causes of recurrent subglottic stenosis. This condition is rare in the literature and prompts more investigation and acquisition of cases to understand its etiology, which could benefit medical management and limit complications.
